# New Validated Short Questionnaire for the Evaluation of the Adherence of Mediterranean Diet and Nutritional Sustainability in All Adult Population Groups

**DOI:** 10.3390/nu14235177

**Published:** 2022-12-05

**Authors:** Stefania Ruggeri, Pasquale Buonocore, Tiziana Amoriello

**Affiliations:** Council for Agricultural Research and Economics (CREA), Research Centre for Food and Nutrition, Via Ardeatina 546, 00178 Rome, Italy

**Keywords:** mediterranean diet, sustainability, adherence, questionnaire, validation study

## Abstract

High adherence to a Mediterranean diet (MD) is favourable for its sustainability and beneficial effects on health. The available questionnaires, according to the MD dietary pattern, include the assessment of moderate alcohol consumption; but some groups, such as young adults and pre-conceptional and pregnant women, are not allowed to consume it. The aim of this study was to validate a new short questionnaire (MedQ-Sus) excluding alcohol consumption, to measure the adherence to the MD and to evaluate the nutritional adherence to a sustainable diet. The Harvard validated questionnaire was used for the validation study. A total of 316 subjects (20 to 70 YOA) completed both questionnaires. A high Spearman correlation coefficient (rho = 0.69; *p* < 0.01) was found between the MedQ-Sus and Harvard scores; a statistically significant positive correlation was found for all eight food groups. The MedQ-Sus had a significant discriminative capacity between adherence and non-adherence to the MD (optimal cut-off point = 9.5, sensitivity 0.86, specificity = 0.65). A very high nutritional adherence to a sustainable diet was found in the subjects for olive oil (97%), dairy food (90%), fresh vegetables (89%), fish and fish products (73), fresh fruit (56%), and cereals and cereals products (42%). A very low adherence was found for legumes (22%) and meat and meat products (9%). The results showed MedQ-Sus is a valid and quick assessment instrument for the evaluation of the adherence to the MD in all population groups, and could also be useful to evaluate the nutritional sustainability of the diet.

## 1. Introduction

The Mediterranean diet (MD) is acknowledged worldwide to be among the best healthy dietary patterns; numerous researchers have demonstrated its beneficial effects for the prevention of many non-communicable diseases, such as diabetes, obesity, cancer and cardiovascular diseases [1,2,3,4,5,6]. Other scientific evidence has emerged about its role in the decrease of low-grade inflammation, and in improving healthy aging and longevity [7,8,9]. The MD’s preventive role is linked to Adverse Reproductive Outcome risk reduction (e.g., Neural Tube Defects and prematurity); following the MD during the pre-conceptional period and pregnancy is associated with positive outcomes for both maternal and offspring health [10,11,12,13,14]. Moreover, the MD is considered by many authors as an example of a sustainable diet, as its dietary pattern is based on a large consumption of vegetables, with a low environmental impact [15,16,17]. Seasonality, biodiversity and social interaction are its founding elements, enabling it to ensure land protection, heritage and the development of traditional activities [18,19,20], as the pattern of a sustainable diet points out [21].

Due to the pivotal beneficial effects of the MD on human health, over the past years many studies have set up questionnaires for the evaluation of the adherence to the MD in epidemiological studies [22,23,24,25,26,27]. It emerged from the systematic review of Zaragoza [27] that few questionnaires [28,29,30] suited the “quality criteria” suggested by some authors [31,32,33,34], e.g., the validity and a lack of data regarding reliability and transcultural adaption (i.e., translation and back translation). Furthermore, the majority of them are time consuming given the number of questions, and not useful for clinical and epidemiological studies in which the use of a much longer questionnaire is not viable.

More recently, some authors validated shorter questionnaires to measure the adherence to the MD, which used very few questions, were less time consuming, and were very useful for clinical practice and/or surveys when time was of the essence [35,36,37,38]. These questionnaires allowed a keen control of compliance in intervention studies, and some of them were tailored for specific population groups, e.g., children, the elderly, cardiovascular patients and pregnant women [39,40,41,42,43].

According to the standard MD dietary pattern [18,44,45], all questionnaires take into account alcohol consumption, giving the highest score for the consumption of 1–2 alcohol units per day/per person (both for men and women) [36,37], > 0 > 1 alcohol units per day (women) and 1–2 alcohol units per day (men) [38], or for a consumption of >7 alcohol units per week [35,41].

However, in the last ten years, strong evidence has emerged showing how alcohol (even in moderate amount) is an increased risk factor for some cancers, such as breast cancer, mouth, pharynx, oesophageal and colon rectal cancer, with the exception of kidney cancer, where up to 30 g per day of alcoholic drinks seems to have a protective role [46]. Exposure to alcohol is one of the major risk factors for the Global Burden of Disease [47,48,49], and unfortunately its consumption is increasing worldwide [50], even among the youngest part of the population—children, adolescents, and young adults [51,52]—with extremely dangerous outcomes mainly on brain development [53,54]. Furthermore, alcohol intake during pregnancy causes foetal alcohol syndrome (FAS) [55,56] with a broad range of conditions, such as neurodevelopmental problems and psychosocial consequences [57,58], and other adverse effects such as an increased risk of miscarriage and prematurity [59,60].

For all these reasons, the majority of dietary recommendations advise adults of legal drinking age to limit alcohol or drink in moderation (usually to two drinks or less in a day for men and one drink or less in a day for women), and pregnant women, children and adolescents to abstain from alcohol [61,62,63,64].

On the basis of all the above, the present study aimed first to validate a new short questionnaire for the evaluation of the MD, without taking into account alcohol consumption, for all adult population groups (young adults 18 to 21 years old, and pre-conceptional and pregnant women included), comparing it with a semi-quantitative questionnaire [65]. Furthermore, the same questionnaire was considered for diet sustainability evaluation according to the food pattern suggested by Willet et al. [21].

## 2. Materials and Methods

### 2.1. Study Population

For the validation study, nutritionists of the National Health Service of 11 Italian regions (Piedmont, Lombardy, Tuscany, Latium, Umbria, Abruzzi, Basilicata, Campania, Apulia, Sardinia and Sicily) invited 500 consecutive healthy subjects from 20 to 74 years of age, between March 2016 and January 2019, to participate in this study in the framework of a partnership with our research centre. The interested subjects were given an informed consent form and the two questionnaires (MedQ-Sus questionnaire and an Italian version of Harvard [65]), with instructions to report their last month’s usual diet. The institutional review boards of the participating institutions approved the study protocol. Each participant was informed about the study and agreed to participate in the data collection and analysis for research purposes alone. Participants’ personal data were anonymous to keep and protect confidentiality. This study was in agreement with the Declaration of Helsinki [66], and national and international regulations.

### 2.2. MedQ-Sus Questionnaire

On the basis of the questionnaire for the evaluation of the adherence to the MD [36], we elaborated a new short questionnaire—the MedQ-Sus questionnaire—without taking into account alcohol consumption for all adult population groups, including young adults (from 18 to 21 years old), pre-conceptional and pregnant women. We decided not to assign any score to alcohol consumption, as it would compromise the results of the adherence total score. This choice allowed us to also use this questionnaire for the evaluation of nutritional sustainability, as alcohol consumption is not contemplated in the food pattern of a sustainable diet [21]. Other small modifications to Sofi’s questionnaire [36] were made on the different kinds of foods and portions. We addressed some food groups in a more precise way (“fresh fruit” instead of “fruit” alone, etc.) and assigned different quantities for oil consumption, to also use this questionnaire for the evaluation of nutritional sustainability (Table 1).

Our new questionnaire included 19 questions divided into 2 sections: (1) Sociodemographic and anthropometric characteristics of subjects (Appendix A). (2) Eight questions on Eight food groups’ consumption: cereals and cereal products, legumes, fresh vegetables, fresh fruit, dairy products, fish and fish products, meat and meat products, and olive oil (Table 1).

In order to evaluate the adherence to the MD, each food group was assigned a quantitative score (from 0 to 2), as showed in Table 2, according to the characteristics of the MD [67] and similar to Sofi [36]. The total MD score ranges from 0 (no adherence) to 16 (high adherence) and is divided into three classes on the basis of tertiles of score distribution: low adherence = 0.0 to 9.0, medium adherence = 9.1 to 11.0 and high adherence = 11.1 to 16.0 (Table 2).

### 2.3. Adherence to The Sustainable Diet

The MedQ-Sus questionnaire was also used to evaluate the adherence to the sustainable healthy diet food pattern. We set up a score for each food group with a dummy variable according to the portion reported in the document of the Eat Lancet Commission [21]: 1 = adherent to the level of food; 0 = not adherent to the level of food (Table 3). The voice “olive oil consumption” of MedQ-Sus was compared with the voice “unsaturated fat” [21].

The total sustainable diet score (SUS) ranges from 0 (no adherence) to 8 (high adherence) and was divided into three classes on the basis of tertiles of score distribution: low adherence = from 0.0 to 3.0, medium adherence = from 3.1 to 4.0, high adherence = from 4.1 to 8.0 (Table 3).

### 2.4. Harvard Questionnaire

To validate MedQ-Sus, the Harvard semi-quantitative food frequency questionnaire (FFQ) [65] was used with minor modification. The comparison between the MedQ-Sus questionnaire and the Harvard one was possible due to some arrangements that allowed us to group up the Harvard semi-quantitative data, and then compare them with our questionnaire.

For each food of the Harvard questionnaire, a consumption quantity has been set up based on the consumption frequency, and for some foods (i.e., fruit and vegetables) on the basis of seasonality as well. A total of 108 items concerning generally eaten food were categorized into 8 food classes: fresh fruit, fresh vegetables, legumes, cereals and cereal products, fish and fish products, meat and meat products, dairy products, and olive oil, and then the amounts consumed for each food were added. The amounts/frequency were then compared with the MedQ-Sus ones. Finally, a transcultural adaption (translation, back translation) of this questionnaire was carried out.

### 2.5. Statistical Analysis

Continuous variables were expressed as median and range (minimum and maximum), and categorical variables as percentages. The validity of the MedQ-Sus questionnaire was assessed by measuring the degree of association between the scores of the MedQ-Sus questionnaire and the Harvard questionnaire, [55] using the Spearman non-parametric correlation. The greater the value of the correlation coefficient rho, the bigger the correspondence between the two questionnaires.

The population adherence to the MD was assessed, turning the MedQ-Sus scores into a dichotomous variable (0 = no, 1 = yes) and identifying a threshold value for individuals following or not following this diet. In accordance with previous research [68,69], a cut-off point was established considering the upper tertile of a reference distribution of a validated questionnaire. Therefore, using the score’s distribution from the Harvard questionnaire, the cut-off was set at 12. The discrimination performance was assessed by the Receiving Operating Characteristic (ROC) curve, i.e., the plot of sensitivity (true positive rate) versus 1-specificty (false positive rate). A test with high discrimination performance shows a ROC curve approaching the upper left corner of the plot. The optimal cut-off between sensitivity and specificity for the MedQ-Sus was calculated using the Youden index, whereas diagnostic accuracy was measured by the area under the curve (AUC). The AUC ranges between 0.5 (no discrimination) and 1 (perfect discrimination). A test shows discriminant power if the 95 % confidence interval values of AUC are greater than 0.50.

Logistic regression was used to describe the relationship between the target variable and several covariates (age, sex, BMI), and highlighted the influence of these three parameters on the adherence to the Mediterranean diet on the basis of previously described cut-offs. Odds ratios (OR) and 95 % confidence intervals were estimated for all logistic models. The *p*-values lesser than 0.05 were considered statistically significant.

All statistical analyses were performed by using SPSS statistical software (SPSS, Chicago, IL, USA).

## 3. Results

One hundred and eighty-four subjects (37% of the sample) invited to participate in the study declined their participation or returned incomplete questionnaires, and were excluded. Complete answers to our questionnaire were available for the remaining 316 individuals (participation rates 63.0%). Several characteristics of the participants (79% females and 21% males) are reported in Table 4.

The participants were, on average, young adults (median = 28), with ages between 20 and 74 years. The subjects were highly educated: 60% of respondents had at least a university degree, and 17% had a lower-education degree. Body Mass Index ranged from 13.1 to 50.8 kg/m^2^: 62% of the participants were of normal weight, 18% were overweight, 13% were obese and 7% were underweight. Finally, 59% of the subjects declared to do physical activity and to be smokers.

**Figure 1:**
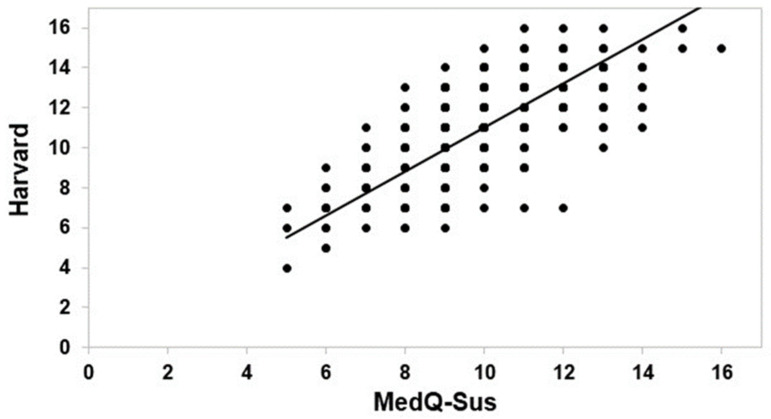
Spearman non-parametric correlation test between total score of Harvard and MedQ-Sus questionnaires.

The correlation between the consumption frequencies for all foods of the Harvard semi-quantitative questionnaire, and the consumption of the corresponding foods from the MedQ-Sus questionnaire, are shown in Figure 1. A good accordance—as coefficients of Spearman (rho = 0.69; *p* < 0.01)—between the two questionnaires was found. The good agreement between the MedQ-Sus and the reference questionnaire indicated the validity of the new method proposed through the MedQ-Sus to measure the adherence to the MD. Moreover, correlation analyses among specific food categories were conducted.

High coefficients of Spearman correlation were found for fresh fruit (rho = 0.64; *p* < 0.01), legumes (rho = 0.70; *p* < 0.01), fish and fish products (rho = 0.57; *p* < 0.01), meat and meat products (rho = 0.58; *p* < 0.01), dairy products (rho = 0.74; *p* < 0.01) and olive oil (rho = 0.99; *p* < 0.01). A discrete correlation was evaluated for fresh vegetables (rho = 0.45; *p* < 0.01), and a low one for cereals and cereal products (rho = 0.14; *p* < 0.05).

**Figure 2:**
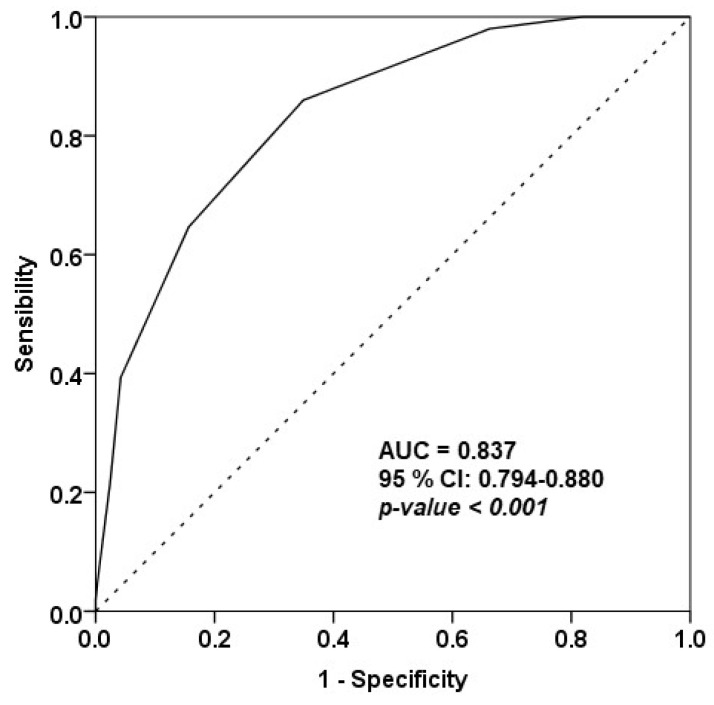
The Receiving Operating Characteristic (ROC) curve for MedQ-Sus questionnaire (AUC = area under the curve, 95 % CI = 95 % confidence interval).

The ROC analysis indicated good discriminative power of the MedQ-Sus questionnaire. The area under the ROC curve is a useful measure to summarize the ROC curve, with a value of AUC = 0.837 and a 95% confidence interval equal to 0.794–0.880 (Figure 2). Being that the AUC value was close to 1, the MedQ-Sus showed good ability to discriminate subjects adhering or not adhering to the MD.

The optimal cut-off calculated using the Youden index was 9.5, with a corresponding sensitivity of 0.86 and specificity of 0.65. However, a considerable number of participants totalized a score between 9 and 10, indicating a possible overlap between adherents and non-adherents to the MD. The best discriminant cut-off between adherents and non-adherents to the MD for the MedQ-Sus questionnaire was 9.5. Therefore, taking into account this threshold, the number of subjects following the MD was 123 (39%), while those not following it numbered 193 (61%). Eventually, multiple logistic regression showed no relationship between the covariates (age, sex and BMI) and the adherence to the MD, as deduced by the non-significance of the odds ratios related to age, sex and BMI (Table 5).

Therefore, the adjustment for these three parameters did not result in any changes in the analysis.

**Figure 3:**
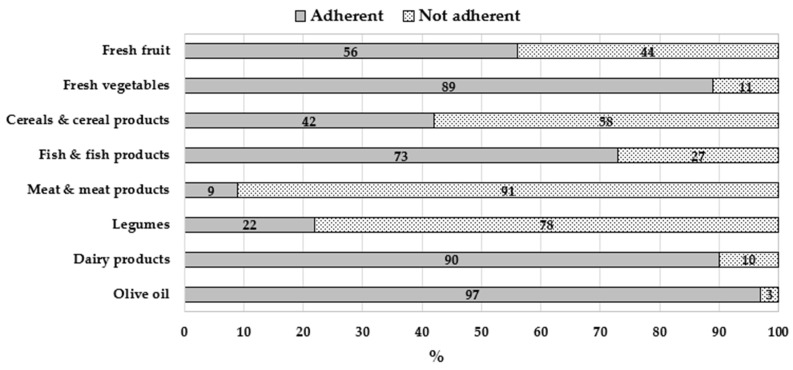
Percentages of individuals following a healthy sustainable diet [21] by food groups, calculated with the MedQ-Sus questionnaire.

The MedQ-Sus questionnaire was also used to evaluate the adherence to the sustainable healthy-diet food pattern [21]. In order to estimate the nutritional adherence to the sustainable diet [21], food group scores were evaluated (Figure 3).

Subjects in this study showed a very high nutritional adherence to the food groups of the sustainable diet [21], mainly concerning olive oil (97%), dairy food (90%) and fresh vegetables (89%), followed by fish and fish products (73%) and fresh fruit (56%). For cereals and cereal products, our sample showed a low adherence (42%); a very low adherence was detected for legumes (22%) and for meat and meat products (9%), the main vegetable and animal protein sources, respectively.

Interesting results emerged from the evaluation of the sustainable total score: 38% of the respondents showed a low adherence to the sustainable-diet nutritional pattern, 34% showed a medium adherence and 28% a high adherence. Concerning the adherence to the MD, 41% of the sample showed a low adherence, 38% a medium adherence and 21% a high one.

## 4. Discussion

In this study, we carried out the validation of a new short questionnaire for the evaluation of the adherence to the MD in all adult population groups, including pre-conceptional and pregnant women, and young adults, who are not allowed to consume alcohol beverages. The need for a new Mediterranean Diet score arose from some considerations in the most recent literature, firstly on the removal of alcohol consumption in the MD score evaluation. Wine is traditionally consumed in Mediterranean countries [25,70], and therefore was included in the first MD food pattern [44,45]. However, as discussed above, emerging evidence demonstrated alcohol’s unhealthy effect on cancer risk increase, even in moderate consumption [46,71]. Furthermore, the benefits on cardiovascular diseases are controversial, depending on the exposure measurement and cardiovascular disease (CVD) outcome as highlighted by several authors [72,73,74,75]. For alcohol-drinking people, the lesser the better.

Recently, the MD dietary pattern concerning alcohol has been revised [18] as well, compared to the original one [25]; the latest MD food pyramid, in fact, does not address a specific amount for alcohol consumption, suggesting otherwise: “wine in moderation and respecting social beliefs” [18].

Thus, the new score suggested in this work took into account the updated version of the MD pyramid by excluding wine [18]. Furthermore, concerning alcohol evaluation, there are some discrepancies among the plethora of questionnaires for MD adherence in adults, as underlined by Aljuraiban et al. [76]. Some questionnaires request alcohol or alcoholic beverage consumption without any indication of sources, including wine and other alcoholic beverages (i.e., soft drinks, spirits) expressed as ethanol grams/day or as alcoholic units [22,23,36]. Other questionnaires, on the contrary, are more adherent to the original MD pattern on wine (number of glass/day or g/day) [25,35,38,41]. These discrepancies could significantly influence the total MD score, as suggested by Eckl et al. [77].

Concerning the evaluation of the adherence to the MD during pregnancy, epidemiological studies showed different approaches. Some have altered the original designs of a priori indexes, scoring alcohol as a detrimental component [78]. Others have removed it entirely from the index [79,80,81]. Excluding alcohol from their index, or reversing its score, contributed to a decreased adherence to the MD, causing a difficult comparison between the studies on pregnant women [77].

For our validation study, we considered a validated questionnaire [36,37] to start, and we improved it by not considering alcohol consumption and by addressing food in a more accurate way (“fresh fruit” instead of “fruit”, “fresh vegetables” instead of “vegetables”) in order to avoid the inclusion of non-healthy processed food (likely rich in sugar and salt), contrary to other questionnaires for the evaluation of the adherence to MD [36,37]. Concerning this issue, some authors [82] underlined that some available questionnaires over-estimated the consumption of some foods like fruits and vegetables, and consequently, the estimated MD scores of the participants may have been imprecise.

With regards to the “cereals” item, we included wholegrain cereals due their beneficial effects on human health [83,84], as suggested in the last MD pyramid [18]. Furthermore, we assigned a quantity for oil consumption and some portion changes. This was fundamental for the evaluation of the nutritional sustainability of the diet, considering the Willet et al. pattern [21].

The MedQ-Sus questionnaire can sort out all the above-mentioned issues, be a very good instrument for the evaluation of the adherence to the MD in all adult groups, and allow an easy comparison of data between epidemiological and clinical studies, given the few questions and the easiness/speediness of administration.

To validate our new questionnaire, we used the semi-quantitative questionnaire by Willet et al. [65] as a reference for food intake evaluation. This questionnaire includes 107 food items, more than Trichopoulou et al. [22], and has often been used in many validation studies [26,27]. We updated some foods in relationship to the current food availability, and a transcultural adaption of the questionnaire was carried out (translation, back translation)—something lacking in many validation studies, as pointed out by Zaragoza et al. [27].

Subjects in this study were recruited voluntarily in the framework of a partnership between our research centre and several nutritionists from the Italian National Health Services, and the study administered during medical examination. For these reasons, the subject number was not very high, but adequate for our scope: to test a new methodology, to evaluate the adherence to the MD in healthy people, and to validate and suggest a new and quick questionnaire. Other studies with similar purposes had comparable or smaller subject numbers in comparison with our study [37,38,85,86]. Moreover, the Italian population is a good case study because Italy is one of the countries recognized as the motherland of the MD [87], where several studies have already been carried out [23,37,38].

The validation showed good results. We found a good correlation between MedQ-Sus total scores and the Harvard questionnaire (rho = 0.69; *p* < 0.01), similar to those obtained from the other study [37], and higher than those obtained in other validation studies tested against food frequency questionnaires [29,38,41]. As underlined by Zaragoza et al. [27], many questionnaires aimed at the evaluation of the adherence of the MD, but only some studies [29] reported information about reliability.

The agreement between the eight food groups of the MedQ-Sus questionnaire and the Harvard ones was very good for some food groups, such as olive oil (rho 0.99 *p* < 0.01), dairy products (rho = 0.74; *p* < 0.01) and legumes (rho = 0.70; *p* < 0.01); lower agreement was found for fresh fruit (rho = 0.64; *p* < 0.01), fish and fish products (rho = 0.57; *p* < 0.01), meat and meat products (rho = 0.58; *p* < 0.01), and fresh vegetables (rho = 0.45; *p* < 0.01), and quite a low one was found for cereals and cereal products (rho = 0.14; *p* < 0.05). This last low value should be ascribed to the fact that the Harvard questionnaire includes more items for some food groups; concerning cereals, the low agreement is probably due to the broad “starchy food group” category in the Harvard questionnaire, which included potatoes and other tubers. However, except for this last food group, the correlation data obtained in our validation study are in the range observed in other studies [23,26], and higher than those observed in studies conducted with a similar methodology [38]. Another good point of our validation study is that no relationship between covariates (age, sex and BMI) and the adherence to the Mediterranean diet was found, even if the distribution was unbalanced (more women than men).

A good discriminative power of the MedQ-Sus questionnaire emerged from the ROC analysis, and the optimal cut-off calculated was 9.5 with a corresponding sensitivity of 0.86 and a specificity of 0.65. These results were similar to those achieved from Martínez-González et al. and Sofi et al. [35,37].

Interesting outcomes arose from the evaluation of diet sustainability; our sample showed good adequacy (with regard to the parameters of a sustainable diet) for many foods and food groups, such as olive oil, dairy food, fresh vegetables, and fish and fish products; and less adequacy, instead, for other groups, such as fresh fruit, and cereals and cereal products.

A remarkable result was the low nutritional adherence to sustainable diet related to two groups: legumes, and meat and meat products, as previously found by the food surveys recently carried out among the Italian population [88]. Results from other recent studies [89,90], based on the Eat Lancet Commission food pattern’s “gold standard” to evaluate nutritional sustainability of diet, showed a similar behaviour regarding high meat and meat product consumption. These results are of great interest, as they focused attention on two food groups, legumes and meat (and their sources of vegetable and animal proteins, respectively), on which communication campaigns must be promoted to improve diet sustainability. The healthy and sustainable diet communication campaigns will have to be strong, suggesting as a solution the increase of legume consumption and the decrease of meat and meat products, as already advised by many authors [89,90,91]. The percentages of adherence to the MD and to sustainable diet gained in our sample (high, medium and low) presented a similar trend, taking into account the limited number of subjects; about two fifths of the subjects showed both low adherence to the MD and to the sustainable diet, probably due to low legume and high meat consumption.

The novelty of this study is a short questionnaire suitable for the evaluation of the adherence to the MD of all adult population groups, including young adults (from 18 to 21 years old), and pre-conceptional and pregnant women. Furthermore, it is the first brief instrument which allows us to simultaneously evaluate the adherence to the MD and the nutritional sustainability of the diet. Despite the fact that this was the first study suggesting a single questionnaire for the evaluation of both adherence and sustainability of the MD, it had some limitations. The first one was the lack of balance among subjects pertaining to sex—the number of male subjects was lower than female ones, as the study has been carried out on a voluntary basis. However, a good heterogeneity regarding education and age was obtained by the recruitment. The second is the fact that the subjects were all Italians and belonged to same ethnic group. Future research should take into account a greater number of subjects and different nationalities and ethnic groups to strengthen our questionnaire, as suggested by the Scientific Committee of the Medical Outcomes Trust [31].

The MedQ-Sus regarding the evaluation of nutritional sustainability showed other limitations as well. Firstly, MedQ-Sus refers to the range portions suggested by Willet et al. [21], for a standard diet of about 2500 calories, thus we will improve it for other caloric intakes. Furthermore, this questionnaire does not consider other fundamental issues of a sustainable diet, e.g., the choice of local, organic and seasonal products, and the evaluation of the carbon and water footprint. Diet sustainability, as highlighted by some authors [92], is a very complex concept, and our future research will aim to improve this questionnaire, adding these other topics.

Lastly, the MedQ-Sus questionnaire provides some advantages, as it is short, very easy to carry out (it is not time consuming), suitable for epidemiological studies and it can be applied in clinical practice to all adults. Furthermore, it allows a better comparison of the adherence to the MD among different population groups (e.g., pregnant women vs childbearing age women, and young adults vs adults) as it does not contemplate alcohol consumption. The MedQ-Sus can also help to understand discrepancies between different food groups and nutritional strategies to increase MD adherence.

## 5. Conclusions

The MedQ-Sus questionnaire is a good instrument to simultaneously evaluate the adherence to the MD and the nutritional sustainability of the diet, and it is very suitable for clinical and epidemiological studies as well.

As the two scores**—**the adherence to the DM and the adherence to the sustainable diet**—**have similar trends in our study, the MedQ-Sus could be a useful instrument for nutritional intervention to improve the adherence to the MD and sustainable food choices.

## Figures and Tables

**Table 1:** Questionnaire for the evaluation of the adherence to the Mediterranean diet in adult population groups.

How Many Portions of These Food Groups Did You Have Last Month?			
Cereals & cereal products (including whole, sweets excluded) 130 g	<1 portion/day	1–1.5 portion/day	>1.5 portion/day
Legumes 70 g	<1 portion/week	1–2 portion/week	>2 portion/week
Fresh vegetables 100 g	<1 portion/day	1–2.5 portion/day	>2.5 portion/day
Fresh fruit 150 g	<1 portion/day	1–2 portion/day	>2 portion/day
Dairy products 180 g	<1 portion/day	1–1.5 portion/day	>1.5 portion/day
Fish & fish products (except shellfish and crustaceans) 100 g	<1 portion/week	1–2.5 portion/week	>2.5 portion/week
Meat & meat products 80 g	<1 portion/day	1–1.5 portion/day	>1.5 portion/day
Olive oil	Occasional Consumption (<5 spoons/day)	Regular Consumption (about 4–5 spoons/day)	Frequent Consumption (>4 spoons/day)

**Table 2:** Mediterranean diet score.

Cereals & cereal products (including whole, sweets excluded)	<1 portion/day 0	1–1.5 portion/day 1	>1.5 portion/day 2
Legumes	<1 portion/week 0	1–2 portion/week 1	>2 portion/week 2
Fresh vegetables	<1 portion/day 0	1-–2.5 portion/day 1	>2.5 portion/day 2
Fresh fruits	<1 portion/day 0	1–2 portion/day 1	>2 portion/day 2
Dairy products	<1 portion/day 2	1–1.5 portion/day 1	>1.5 portion/day 0
Fish and fish products (except shellfish and crustaceans)	<1 portion/week 0	1–2.5 portion/week 1	>2.5 portion/week 2
Meat and meat products	<1 portion/day 2	1–1.5 portion/day 1	>1,5 portion/day 0
Olive oil	Occasional Consumption (<5 spoons/day) 0	Regular Consumption (about 4–5 spoons/day) 2	Frequent Consumption (>4 spoons/day) 1

Mediterranean total score: low adherence = 0 to 9.0; medium adherence = 9.1 to 11.0; high adherence = 11.1 to 16.0.

**Table 3:** Sustainable diet score.

Cereals & cereal products (including whole, sweets excluded)	<1 portion/day 0	1–1.5 portion/day 0	>1.5 portion/day 1
Legumes	<1 portion/week 0	1–2 portion/week 0	>2 portion/week 1
Fresh vegetables	<1 portion/day 0	1–2.5 portion/day 1	>2.5 portion/day 1
Fresh fruits	<1 portion/day 0	1–2 portion/day 1	>2 portion/day 0
Dairy products	<1 portion/day 0	1–1.5 portion/day 1	>1.5 portion/day 0
Fish and fish products (except shellfish and crustaceans)	<1 portion/week 0	1–2.5 portion/week 1	>2.5 portion/week 1
Meat and meat products	<1 portion/day 1	1–1.5 portion/day 0	>1.5 portion/day 0
Olive oil	Occasional Consumption (<5 spoons/day) 0	Regular Consumption (about 4–5 spoons/day) 1	Frequent Consumption (>4 spoons/day) 0

Sustainable total score: low adherence = 0.0 to 3.0; medium adherence = 3.1 to 4.0; high adherence = 4.1 to 8.0.

**Table 4:** Characteristics of the study participants (n = 316).

Characteristics	Value
Age (median, range)	28 (20–74)
Sex	
Male (%)	21
Female (%)	79
Education (years)	
5 (%)	4
8 (%)	13
13 (%)	23
16 (%)	38
18 (%)	19
>18 (%)	3
Weight (kg) (median, range)	61 (40–140)
Height (m) (median, range)	1.65 (1.50–1.93)
Body mass index (kg/m^2^) (median, range)	22.6 (13.1–50.8)
Body mass index	
Low weight (%)	7
Normal weight (%)	62
Overweight (%)	18
Obesity (%)	13
Smoking habits	
Yes (%)	59
No (%)	41
Physical activity	
Yes (%)	59
No (%)	41

**Table 5:** Multiple logistic regression analysis and odds ratios (OR) with 95 % confidence intervals for a diagnosis of adherence to the Mediterranean diet.

	OR	95% CI	*p*-Value
Age	0.993	0.971–1.015	0.523
Sex	1.825	0.990–3.365	0.054
BMI	0.957	0.905–1.012	0.120

## Data Availability

The data presented in this study are available on request from the corresponding author.

## Appendix A

Subject sociodemographic and anthropometric characteristics.

**Subject number**	
Sex	○ Male ○ Female
How old are you?	
In which country do you live?	_________________________________
In which city?	_________________________________
What is your current status?	○ Single ○ In a relationship or living with my partner ○ Married ○ Divorced ○ Widowed ○ Other
What is your level of education?	
Employment	○ Housewife ○ Student ○ Employed ○ Self- employed ○ Unemployed ○ other
How much do you weight? (kg)	
How tall are you? (cm)	
Do you smoke?	○ Yes ○ No
Do you enjoy physical activity?	○ Yes ○ No If yes, please describe indetail
issue date	
